# AT_1_ receptor blocker, but not an ACE inhibitor, prevents kidneys from hypoperfusion during congestive heart failure in normotensive and hypertensive rats

**DOI:** 10.1038/s41598-021-83906-6

**Published:** 2021-02-19

**Authors:** Vojtech Kratky, Zdenka Vanourkova, Matus Sykora, Barbara Szeiffova Bacova, Zdenka Hruskova, Sona Kikerlova, Zuzana Huskova, Libor Kopkan

**Affiliations:** 1 Center for Experimental Medicine, Institute for Clinical and Experimental Medicine, 1958/9 Videnska, 14000 Prague 4, Czech Republic; 2 Department of Pathophysiology, 2nd Faculty of Medicine, Charles University, Prague, Czech Republic; 3 Department of Nephrology, First Faculty of Medicine, Charles University and General University Hospital in Prague, Prague, Czech Republic; 4 Institute for Heart Research, Centre of Experimental Medicine, Slovak Academy of Sciences, Bratislava, Slovak Republic

**Keywords:** Circulation, Kidney

## Abstract

To provide novel insights into the pathogenesis of heart failure-induced renal dysfunction, we compared the effects of ACE inhibitor (ACEi) and AT_1_ receptor blocker (ARB) on systemic and kidney hemodynamics during heart failure in normotensive HanSD and hypertensive transgenic (TGR) rats. High-output heart failure was induced by creating an aorto-caval fistula (ACF). After five weeks, rats were either left untreated or treatment with ACEi or ARB was started for 15 weeks. Subsequently, echocardiographic, renal hemodynamic and biochemical measurements were assessed. Untreated ACF rats with ACF displayed significantly reduced renal blood flow (RBF) (HanSD: 8.9 ± 1.0 vs. 4.7 ± 1.6; TGR: 10.2 ± 1.9 vs. 5.9 ± 1.2 ml/min, both *P* < .001), ACEi had no major RBF effect, whereas ARB completely restored RBF (HanSD: 5.6 ± 1.1 vs. 9.0 ± 1.5; TGR: 7.0 ± 1.2 vs. 10.9 ± 1.9 ml/min, both *P* < .001). RBF reduction in untreated and ACEi-treated rats was accompanied by renal hypoxia as measured by renal lactate dehydrogenase activity, which was ameliorated with ARB treatment (HanSD: 40 ± 4 vs. 42 ± 3 vs. 29 ± 5; TGR: 88 ± 4 vs. 76 ± 4 vs. 58 ± 4 milliunits/mL, all *P* < .01). Unlike improvement seen in ARB-treated rats, ACE inhibition didn’t affect urinary nitrates compared to untreated ACF TGR rats (50 ± 14 vs. 22 ± 13 vs. 30 ± 13 μmol/mmol Cr, both *P* < .05). ARB was more effective than ACEi in reducing elevated renal oxidative stress following ACF placement. A marker of ACEi efficacy, the angiotensin I/angiotensin II ratio, was more than ten times lower in renal tissue than in plasma. Our study shows that ARB treatment, in contrast to ACEi administration, prevents renal hypoperfusion and hypoxia in ACF rats with concomitant improvement in NO bioavailability and oxidative stress reduction. The inability of ACE inhibition to improve renal hypoperfusion in ACF rats may result from incomplete intrarenal RAS suppression in the face of depleted compensatory mechanisms.

## Introduction

An enormous rise in the prevalence of heart failure (HF) is causing a tremendous burden on healthcare systems worldwide, and HF is now considered as a global pandemic^1^. Currently, HF can be divided into heart failure with reduced ejection fraction (HFrEF), heart failure with preserved ejection fraction (HFpEF) and somewhat controversial heart failure with mid-range ejection fraction (HFmrEF)^2^. This division is based on the left ventricular ejection fraction (LVEF), while patients with LVEF < 40% are classified to have HFrEF, patients with LVEF 40–49% HFmrEF and patient with LVEF ≥ 50% HFpEF. Although many advances were made in developing effective treatment strategies for HFrEF patients in the past decades, an evidence-based mortality-lowering therapeutic protocol is still missing. Therefore, there is a great need for a more in-depth understanding of HFpEF pathophysiology, that would ultimately lead to an improvement in management and therapy of patients with HFpEF^3^. The kidney is one of the most important organs involved in the progression of HF. There are numerous heart-kidney interactions that lead to the development of kidney dysfunction during chronic heart failure^4,5^. And since kidney functions are an important predictor of mortality in HF^6^, there is a consensus that we can improve the prognosis of patients with HF by preventing the development of renal dysfunction^7^.

Chronic heart failure is not entirely only a hemodynamic disorder but also activates important compensatory systems that help to counterbalance reduced heart functions. However, excessive activation of these systems is in the long term detrimental^8^. The renin-angiotensin system (RAS) and the sympathetic nervous system (SNS) are two of the most critical systems that play a role in HF progression. Especially in the kidney, RAS and SNS activation triggers a number of responses that negatively influence the ability of the kidney to appropriately maintain electrolyte and body fluid balance^9^. Most widely used drugs to inhibit RAS are angiotensin type 1 (AT_1_) receptor blockers and angiotensin-converting enzyme (ACE) inhibitors. By reducing the effects of angiotensin II (ANG II), the most important peptide of the RAS cascade, they directly influence not only blood pressure but also vascular function and thus organ hemodynamics. Circulating ANG II and most likely local tissue ANG II generation affect several mechanisms involved in the response of heart and kidneys to HF-induced injury. There is large evidence of RAS and SNS crosstalk on both local and systemic level^10^. ANG II is also a known activator of several signaling molecules in multiple downstream pathways, including kinases, transcription factors, cytokines, and growth factors, and modulates activity of reactive oxygen species (ROS) or nitric oxide (NO) production^11^. Thus, the inhibitors of the RAS significantly influence these processes and display important protective actions to the heart and kidney functions.

Although both AT_1_ receptor blockers and ACE inhibitors are considered to be as equally powerful in the treatment of HF, there are crucial differences in responses of the RAS to their action^12^. So far, a direct comparison of long-term treatment with these two classes of drugs in rats with aorto-caval fistula (ACF), a well-established model of volume-overload induced heart failure^13^, is missing. Since activation of RAS is a common finding in HF and hypertension is one of the major risk factors for the detrimental progression of HF, the hypertensive Ren-2 transgenic rat (TGR) with ACF is a reliable model of aggravated high-output heart failure^14^. Thus, a major aim of this study was to compare the effect of ACE inhibitors and AT_1_ receptor blockers on systemic and particularly kidney hemodynamics during heart failure in normotensive and hypertensive rats.

## Results

Table 1 summarizes body weights and ratios of organ weights to tibial lengths in control rats, untreated HF rats, and rats treated with either ACE inhibitor or AT_1_ blocker. Raw organ weights are also shown in Supplementary Table 1. Untreated Hannover Sprague–Dawley (HanSD) and TGR rats with induced heart failure displayed marked cardiac hypertrophy as is apparent from a significant increase in heart weight/tibial length ratios. ACE inhibitor significantly attenuated the hypertrophic response in both strains of rats. However, AT_1_ blocker significantly reduced heart weights only in TGR rats with ACF. Both right and left ventricle contributed to the observed heart weight increase in ACF rats. Lung weight/tibial length ratios were significantly increased in untreated HanSD and TGR rats with ACF when compared to sham-operated rats, indicating HF-induced lung congestion. Treatment with ACE inhibitor and AT_1_ blocker significantly reduced lung congestion in both strains of rats. At 20 weeks after ACF induction, 12 out of 16 untreated (75%) HanSD rats were alive, while there were 11 out of 13 (85 percent) surviving among ACE inhibitor-treated as well as AT_1_ blocker-treated HanSD rats. The percentage of alive ACE inhibitor- or AT_1_ blocker-treated TGR rats with ACF at 20 weeks after ACF operation was similarly as in HanSD rats 86% (data not shown).

**Table 1:** Body weights, tibial lengths and ratios of organ weights to tibial lengths in control, untreated heart failure (HF), ACE inhibitor-treated (HF + ACEi) and AT_1_ receptor blocker-treated (HF + ARB) HanSD and TGR rats.

	HanSD rats				TGR rats			
	Control	HF	HF + ACEi	HF + ARB	Control	HF	HF + ACEi	HF + ARB
Body Weight (g)	543 ± 37	548 ± 45	537 ± 31	575 ± 56	652 ± 46^**¥**^	483 ± 46*	606 ± 53^#¥^	643 ± 38^#¥^
Tibial length (mm)	42.9 ± 1.1	43 ± 0.8	42.8 ± 0.7	43.3 ± 1.1	45 ± 0.6^**¥**^	38.9 ± 1.3*^¥^	44.6 ± 0.7^#¥^	44.8 ± 0.7^#¥^
Heart weight (mg)/Tibial length (mm)	35.2 ± 2.2	64.6 ± 11*	54 ± 5.2*^#^	58.1 ± 5.7*	43.2 ± 2.4^**¥**^	63.6 ± 8.8*	48.1 ± 5.3*^#^	49.1 ± 5.8*^#^
Left ventricle weight (mg)/Tibial length (mm)	25.8 ± 1.5	40.9 ± 5.1*	35.6 ± 3.1*^#^	38.5 ± 3.2*	32.9 ± 1.6^**¥**^	43.5 ± 1.2*	31.7 ± 3.2^#¥^	33.1 ± 1.2^#¥^
Right ventricle weight (mg)/Tibial length (mm)	7.2 ± 0.6	14.5 ± 2.9*	12.2 ± 1.3*^#^	13.2 ± 1.7*	6.9 ± 0.5*	12.7 ± 2.7*	11.6 ± 2.5*	11 ± 1.6*
Lung weight (mg)/Tibial length (mm)	46.2 ± 2.2	61.9 ± 12.9*	52.2 ± 5^**#**^	53.7 ± 6.4^**#**^	45.6 ± 3.5	65.9 ± 14.1*	52.6 ± 4.9^**#**^	52.9 ± 4.3^**#**^
Liver weight (mg)/Tibial length (mm)	392 ± 44	445 ± 132	406 ± 62	391 ± 41	444 ± 22	458 ± 89	404 ± 56	431 ± 44
Kidney weight (mg)/Tibial length (mm)	45 ± 4.5	42.8 ± 1.5	44.8 ± 5.4	43.6 ± 3.6	47.3 ± 1.6	44.8 ± 3.9	43.4 ± 4.3	44.2 ± 2.9

Please note that data from HF TGR rats were obtain in 5 weeks after ACF induction due to high mortality.

Values are expressed as mean ± standard deviation and consist of ten observations per group. *P < 0.05 versus control of the same rat strain ^#^*P* < 0.05 versus HF of the same rat strain ^¥^*P* < 0.05 versus HanSD counterparts.

**Figure 1:**
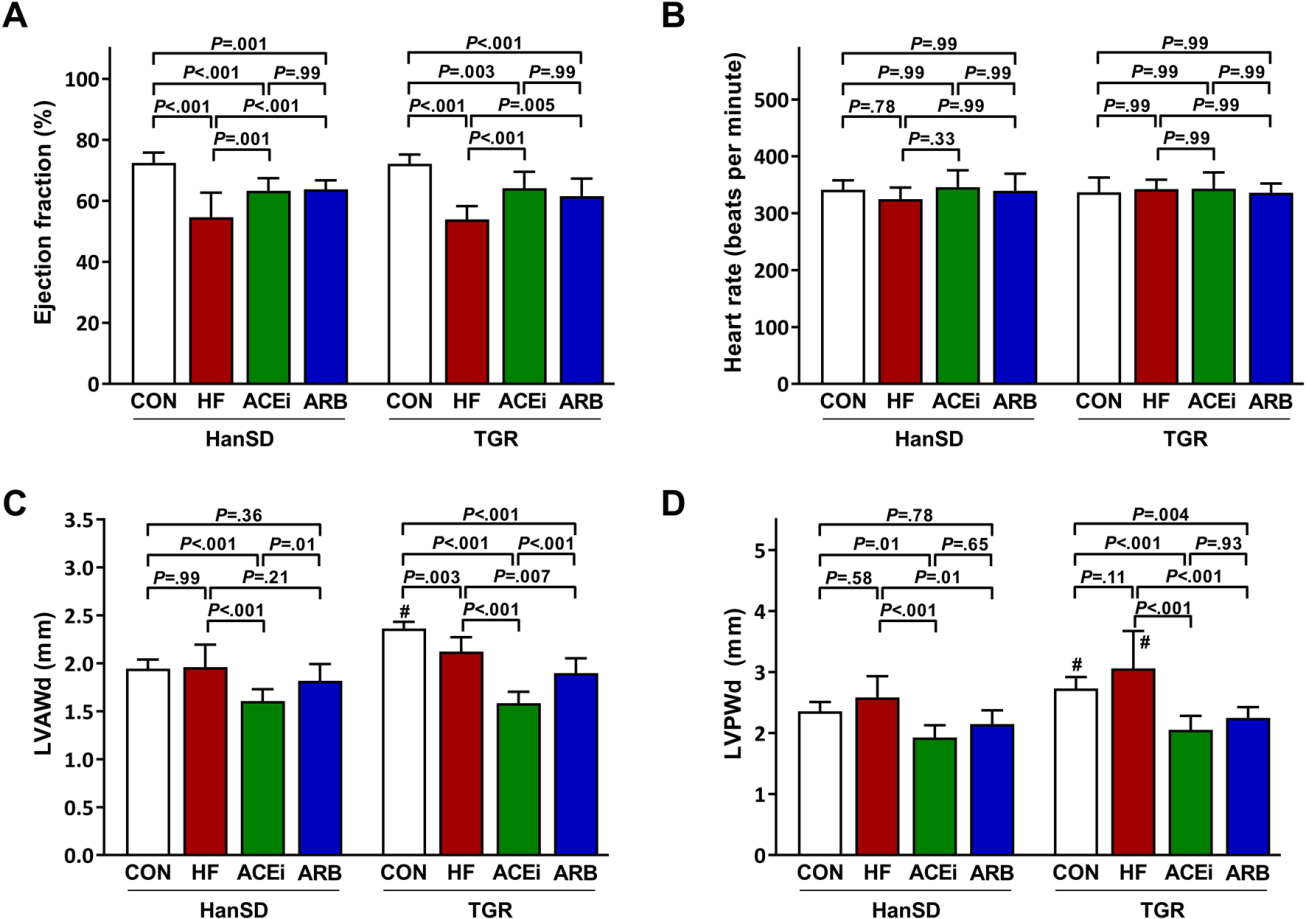
Cardiac ejection fractions (**A**), heart rates (**B**), left ventricle anterior wall dimensions (LWAVd) (**C**) and left ventricle posterior wall dimensions (LVPWd) (**D**) of control (CON), untreated heart failure (HF), ACE inhibitor-treated (ACEi) and AT_1_ receptor blocker-treated (ARB) HanSD and TGR rats 20 weeks after sham or ACF operation (5 weeks in HF TGR rats due to high mortality). Data presented as mean ± SD (n = 10 in each group). ^#^*P* < 0.05 compared to HanSD rats.

Figure 1 shows cardiac parameters assessed by echocardiography. Creation of ACF resulted in a decline in heart function as ejection fractions were significantly lower in untreated HF rats compared to control HanSD rats (54.6 ± 8.1% vs. 72.5 ± 3.4%, *P* < 0.001). Long-term administration of ACE inhibitor or AT_1_ blocker to HF rats significantly improved ejection fraction (63.3 ± 4.1% and 63.8 ± 2.9%, both *P* < 0.01). In addition, TGR rats displayed an identical pattern of changes in heart performance as HanSD rats (Fig. 1A). The observed significant differences in ejection fractions were measured at similar heart rate levels (Fig. 1B). In HanSD rats, left ventricle anterior wall in diastole (LVAWd) and left ventricle posterior wall in diastole (LVPWd) dimensions were not affected by the development of HF (Fig. 1C,D). On the other hand, LVAWd was significantly reduced in TGR rats 5 weeks after ACF operation (Fig. 1C), dissimilar to LVPWd, which remained unaffected by the HF development in TGR rats. Taken together with a marked increase in heart weight in rats with ACF, these findings suggest that the hearts of ACF rats undergo extensive eccentric cardiac hypertrophy, as illustrated in previous studies^15,16^. Both treatments significantly reduced LVAWd and LVPWd dimensions, although the reduction was significantly more pronounced in rats treated with an ACE inhibitor, as shown in Fig. 1C.

**Figure 2:**
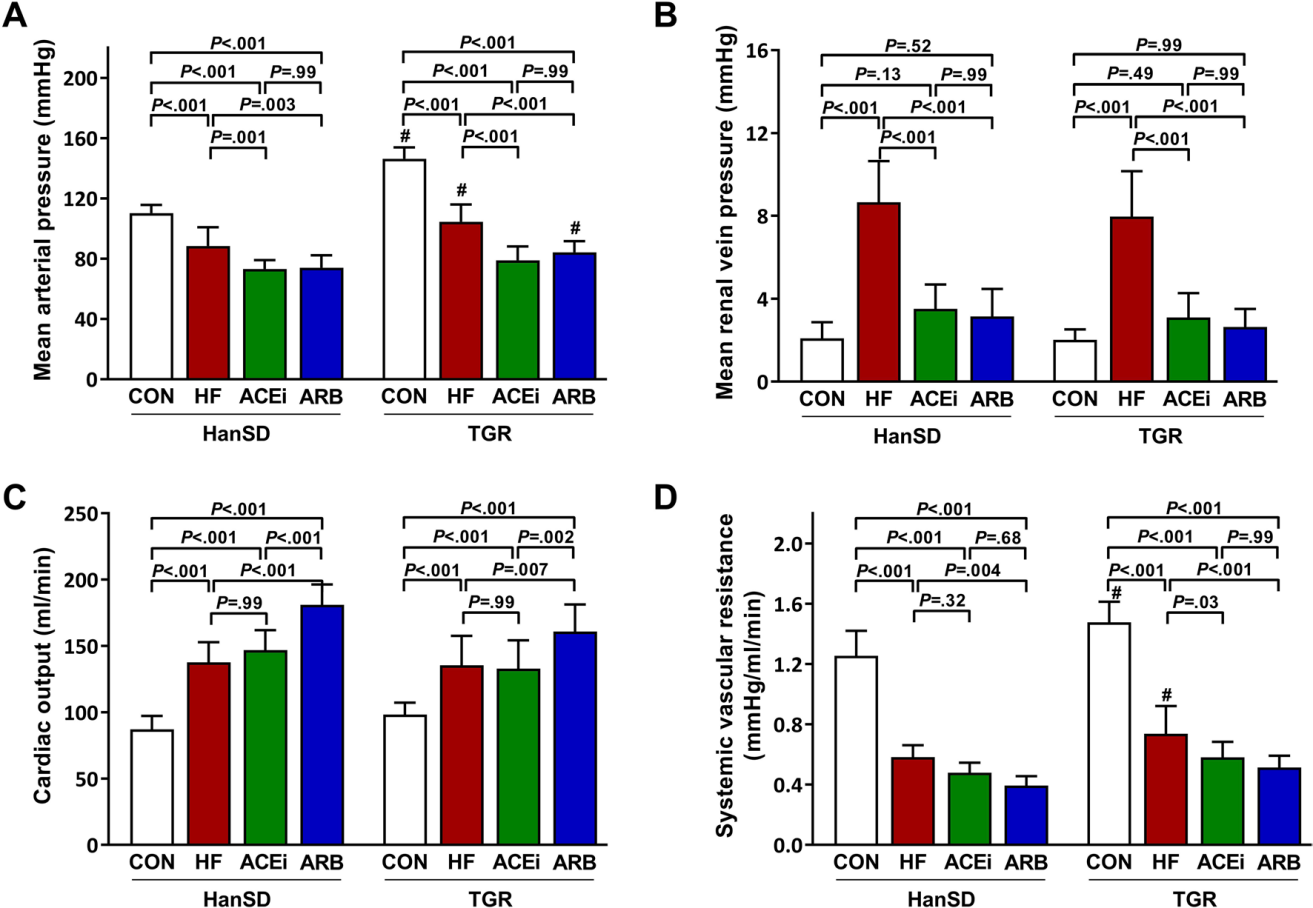
Mean arterial pressures (**A**), mean renal vein pressures (**B**), cardiac outputs (**C**) and systemic vascular resistances (**D**) of control (CON), untreated heart failure (HF), ACE inhibitor-treated (ACEi) and AT_1_ receptor blocker-treated (ARB) HanSD and TGR rats 20 weeks after sham or ACF operation (5 weeks in HF TGR rats due to high mortality). Data presented as mean ± SD (n = 10 in each group). ^#^*P* < 0.05 compared to HanSD rats.

Systemic hemodynamic parameters are summarized in Fig. 2. Mean arterial pressure (MAP) was significantly higher in control TGR rats when compared to HanSD rats (110 ± 5 mmHg vs. 146 ± 8 mmHg, *P* < 0.001). The creation of ACF resulted in significantly decreased MAP in HanSD as well as in TGR rats (88 ± 12 mmHg and 104 ± 11 mmHg, both *P* < 0.001). Inhibition of RAS by either drug led to further MAP reduction to a comparable extent in all experimental groups (Fig. 2A). We observed a fourfold rise in mean renal vein pressure in rats with heart failure compared to control animals (2.1 ± 0.8 mmHg vs. 8.7 ± 2.0 mmHg in HanSD rats, 2.0 ± 0.5 mmHg vs. 8.0 ± 2.2 mmHg in TGR rats, both *P* < 0.001). However, HF rats treated with an ACE inhibitor or AT_1_ receptor blocker had vein pressures in kidneys not statistically different from those in control groups (Fig. 2B). Cardiac output increased in all groups with ACF when compared to control groups, and additionally even more in groups treated with AT_1_ blocker as illustrated in Fig. 2C. Correspondingly, all rats with ACF had substantially decreased systemic vascular resistance (Fig. 2D).

**Figure 3:**
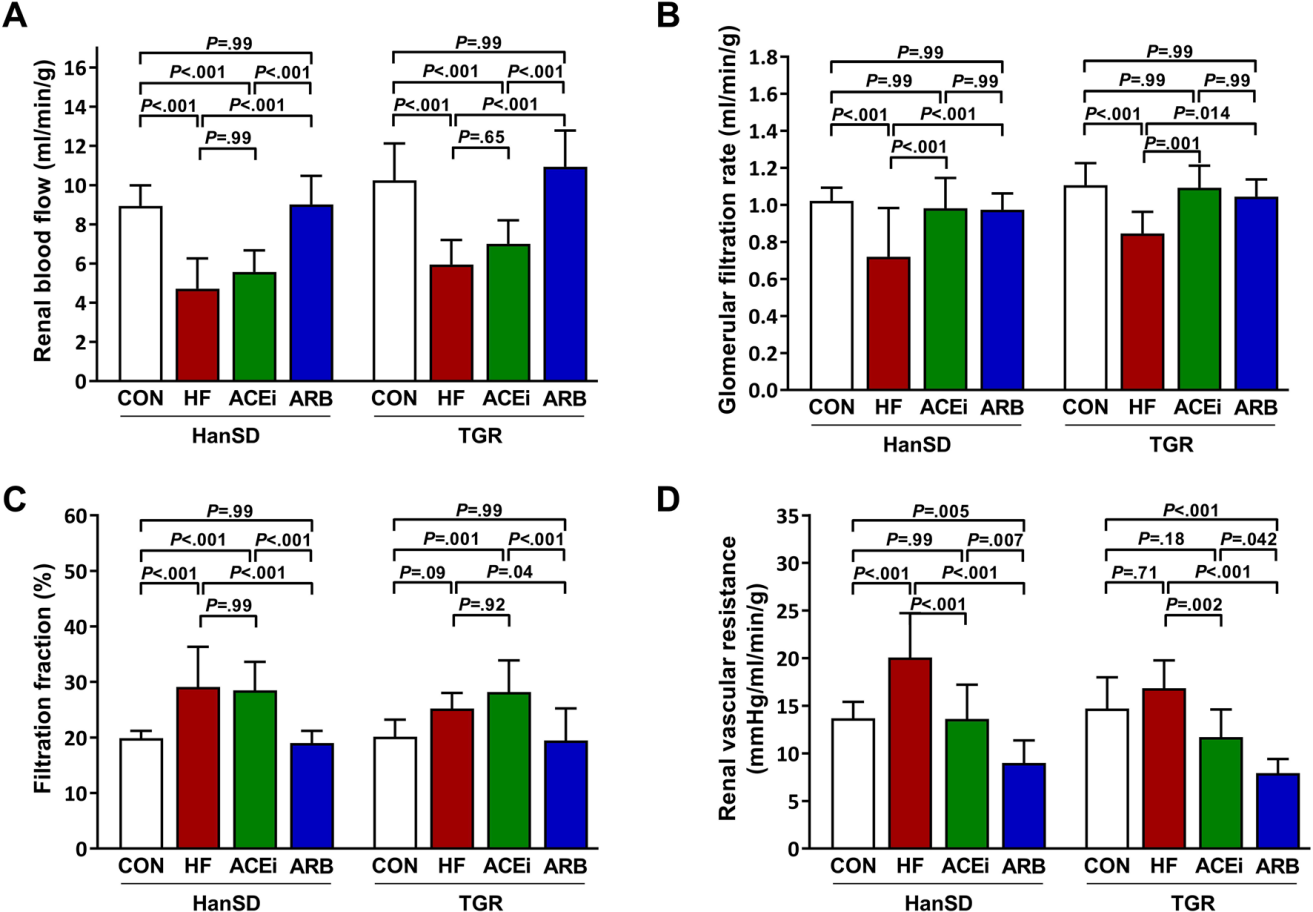
Renal blood flows (**A**), glomerular filtration rates (**B**), filtration fractions (**C**) and renal vascular resistances (**D**) of control (CON), untreated heart failure (HF), ACE inhibitor-treated (ACEi) and AT_1_ receptor blocker-treated (ARB) HanSD and TGR rats 20 weeks after sham or ACF operation (5 weeks in HF TGR rats due to high mortality). Data presented as mean ± SD (n = 10 in each group).

Renal hemodynamic parameters are shown in Fig. 3. Renal blood flow was significantly reduced in animals with HF compared to control HanSD rats (8.9 ± 1.0 ml/min vs. 4.7 ± 1.6 ml/min, *P* < 0.001). Treatment with ACE inhibitor did not significantly affect blood flow through kidneys (5.6 ± 1.1 ml/min), unlike AT_1_ receptor blocker that improved renal blood flow (9.0 ± 1.5 ml/min) as presented in Fig. 3A. We observed an identical pattern of changes in TGR rats. Glomerular filtration rate significantly deteriorated only in untreated rats with ACF, whereas glomerular filtration rates (GFRs) in other groups of animals was equivalent to rates observed in control animals (Fig. 3B). Nonetheless, filtration fraction was significantly higher in untreated HF rats as well as ACE inhibitor-treated rats when compared to control animals. On the other hand, AT_1_ blocker normalized filtration fraction in rats with ACF (Fig. 3C). Renal vascular resistance was significantly higher in untreated rats with ACF than in control rats but administering ACE inhibitor to ACF rats reduced vascular resistance to normal levels. Notably, renal vascular resistance was further significantly decreased in rats treated with AT_1_ blocker when compared to ACE inhibitor-treated rats, and this effect was observed in both HanSD and TGR rats, as demonstrated in Fig. 3D.

**Figure 4:**
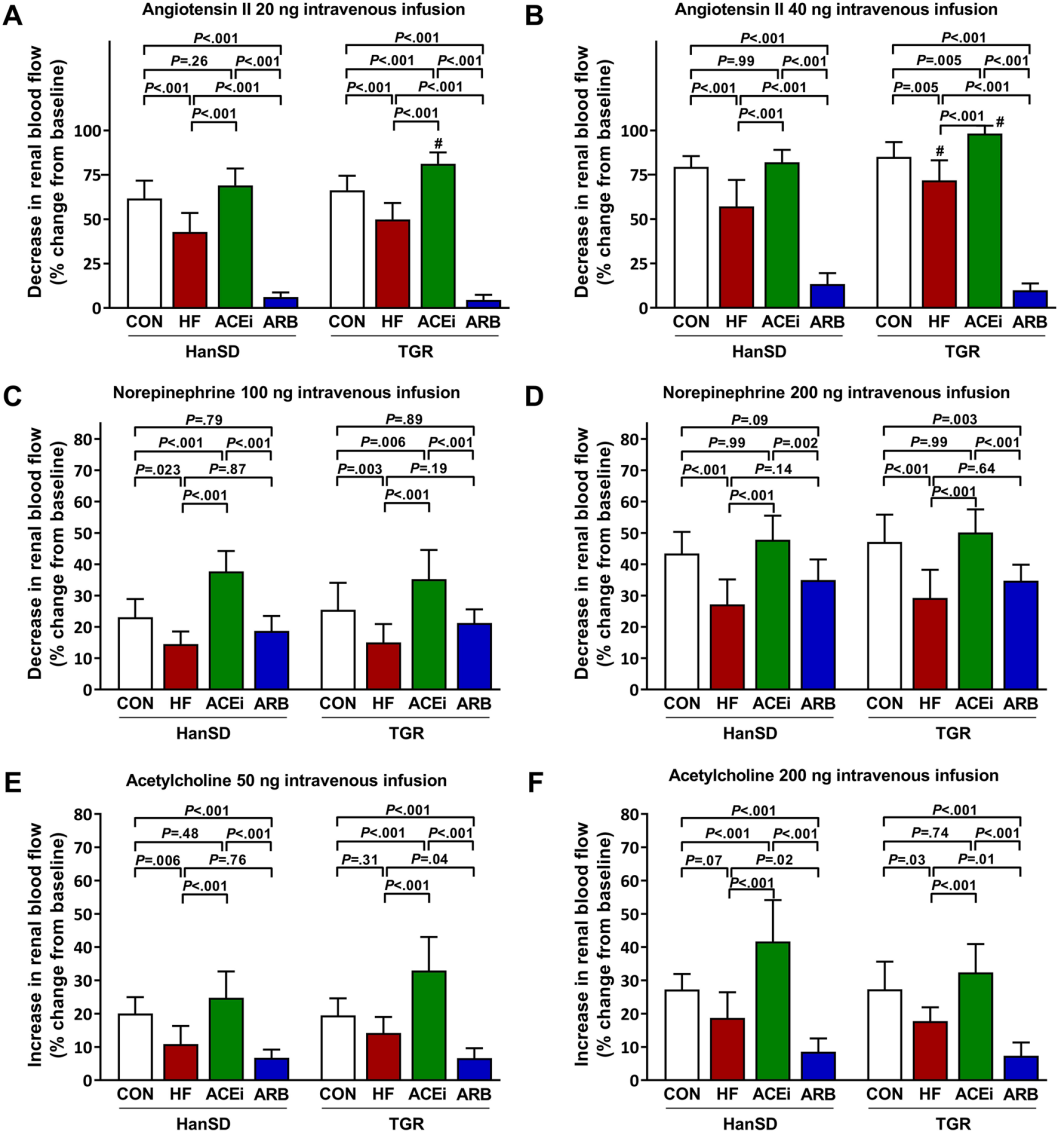
Maximum changes in renal blood flow elicited by intravenous boluses of angiotensin II 20 ng (**A**), angiotensin II 40 ng (**B**), norepinephrine 100 ng (**C**), norepinephrine 200 ng (**D**), acetylcholine 50 ng (**E**) and acetylcholine 200 ng (**F**) of control (CON), untreated heart failure (HF), ACE inhibitor-treated (ACEi) and AT_1_ receptor blocker-treated (ARB) HanSD and TGR rats 20 weeks after sham or ACF operation (5 weeks in HF TGR rats due to high mortality). Data presented as mean ± SD (n = 10 in each group). ^#^*P* < 0.05 compared to HanSD rats.

Figure 4 summarizes responses of the renal vasculature to intravenously infused boluses of ANG II, norepinephrine, and acetylcholine. Rapid infusion of 20 ng and 40 ng of ANG II to ACF rats elicited significantly smaller decreases in renal blood flow (RBF) when compared to control rats. Rats treated with ACE inhibitor had renal vascular responsiveness to ANG II significantly greater when compared to untreated HF rats. Furthermore, TGR rats treated with ACE inhibitor exhibited significantly augmented renal responses to ANG II compared to control animals. As expected, intravenous infusion of ANG II to rats treated with AT_1_ blocker produced only minuscule responses in RBF (Fig. 4A,B respectively). We observed significantly smaller RBF decreases after boluses of 100 and 200 ng of norepinephrine in untreated HF rats when compared to control rats. Despite that, rats treated with an ACE inhibitor displayed substantially larger decreases in RBF after norepinephrine boluses. In contrast with rats treated with an ACE inhibitor, AT_1_ blocker-treated rats had renal vascular responsiveness to norepinephrine comparable to untreated HF rats (Fig. 4C,D respectively). Increases in RBF after intravenous acetylcholine infusion were reduced in untreated ACF rats compared to control animals. However, administering ACE inhibitor to ACF rats led to significantly larger increases in RBF than in untreated animals. On the other hand, responses to 50 and 200 ng of acetylcholine were significantly suppressed in rats given AT_1_ blocker (Fig. 4E,F respectively).

**Figure 5:**
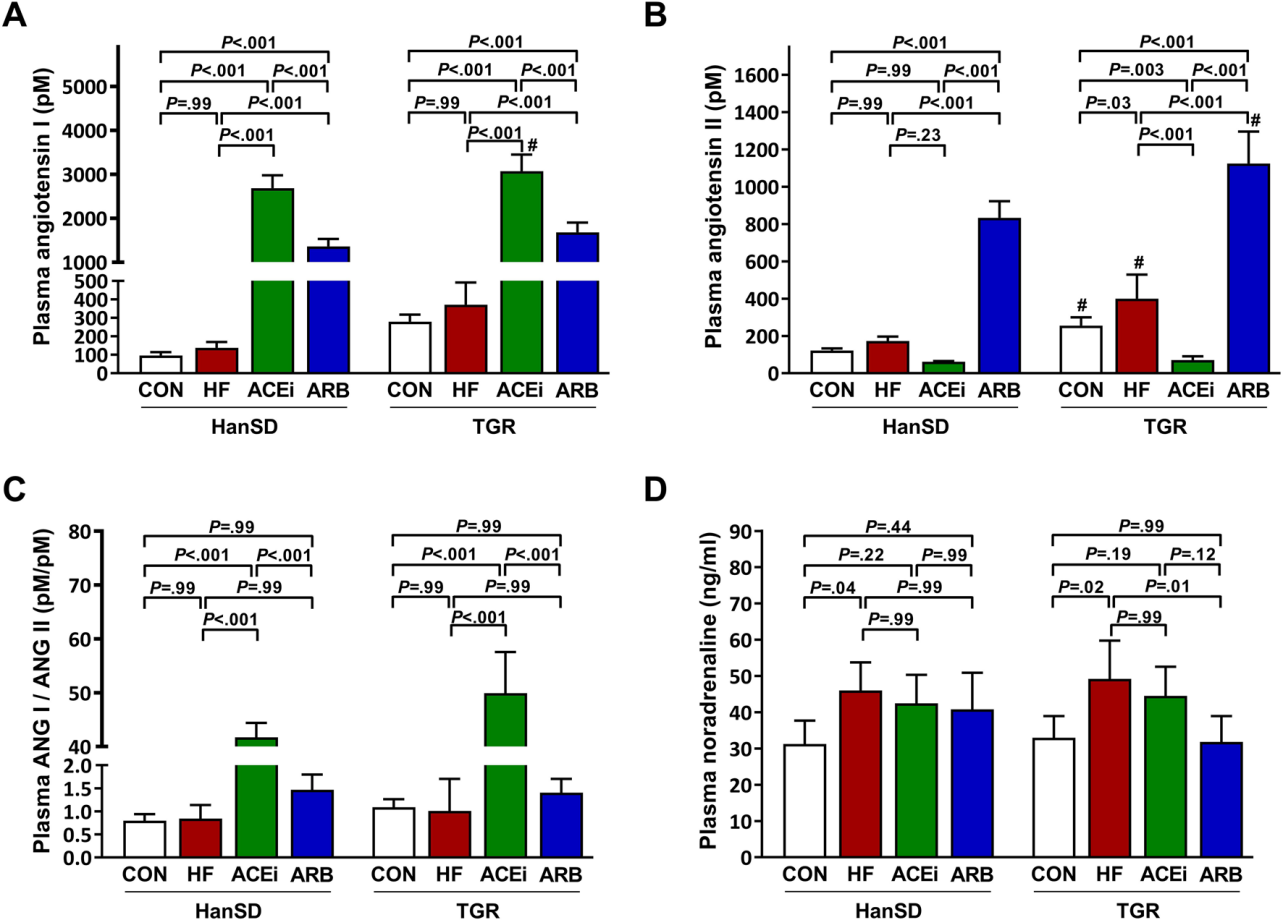
Plasma angiotensin I levels (**A**), plasma angiotensin II levels (**B**), plasma angiotensin I to angiotensin II ratios (**C**) and plasma noradrenaline levels (**D**) of control (CON), untreated heart failure (HF), ACE inhibitor-treated (ACEi) and AT_1_ receptor blocker-treated (ARB) HanSD and TGR rats 20 weeks after sham or ACF operation (5 weeks in HF TGR rats due to high mortality). Data presented as mean ± SD (n = 5–6 in each group). ^#^*P* < 0.05 compared to HanSD rats.

Levels of angiotensins and noradrenaline in plasma are summarized in Fig. 5. Although plasma angiotensin I (ANG I) levels did not significantly differ between control and HF rats, ANG I levels were significantly increased following ACEi or ARB administration (Fig. 5A). As expected, control TGR rats had significantly higher ANG II concentration in plasma compared to HanSD rats. We observed a significant increase in plasma ANG II levels following ACF operation only in TGR rats. Likewise, ACE inhibition significantly suppressed plasma ANG II in TGR rats, but not in HanSD rats. In both rat strains, ARB treatment led to a substantial rise of ANG II in plasma (Fig. 5B). However, the pattern of changes in ANG II levels was similar in HanSD rats as in TGR rats, and the statistical difference was not only reached due to enormous deviation of the ARB group. Figure 5C shows ANG I/ANG II ratios in plasma. In both rat strains, ACE inhibition led to a tremendous rise in plasma ANG I/ANG II ratio, suggesting an adequate ACE suppression. Untreated HanSD and TGR rats with HF exhibited significantly increased plasma noradrenaline levels compared to control animals. In TGR rats with ACF, ARB administration was associated with a significant plasma noradrenaline decrease (Fig. 5D).

**Figure 6:**
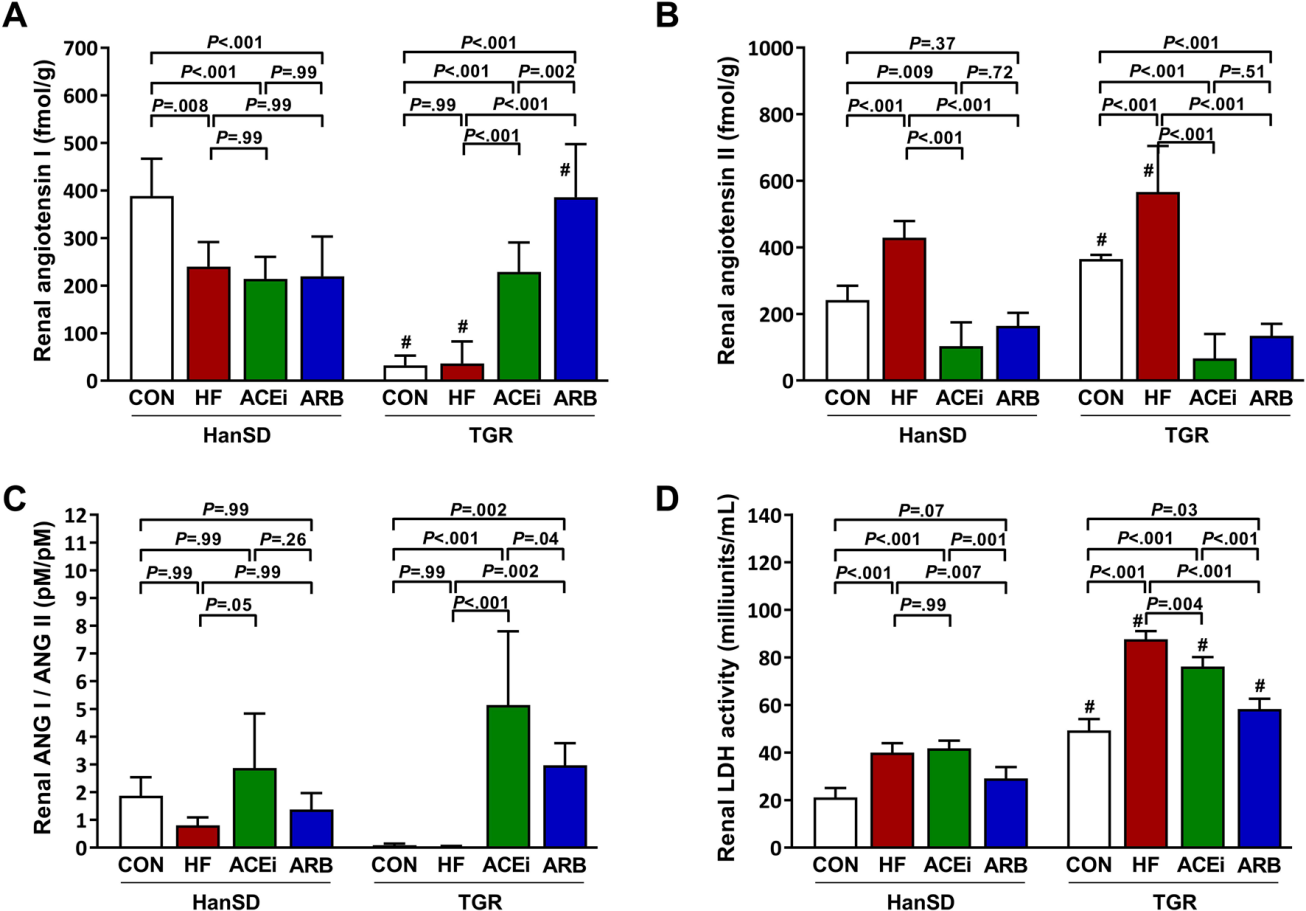
Renal angiotensin I levels (**A**), renal angiotensin II levels (**B**), renal angiotensin I to angiotensin II ratios (**C**) and renal lactate dehydrogenase (LDH) activity (**D**) of control (CON), untreated heart failure (HF), ACE inhibitor-treated (ACEi) and AT_1_ receptor blocker-treated (ARB) HanSD and TGR rats 20 weeks after sham or ACF operation (5 weeks in HF TGR rats due to high mortality). Data presented as mean ± SD (n = 4–6 in each group). ^#^*P* < 0.05 compared to HanSD rats.

Shown in Fig. 6 are renal levels of angiotensins and renal lactate dehydrogenase (LDH) activity. HanSD rats showed significantly reduced renal ANG I content after ACF placement, and neither treatment further influenced ANG I levels in the kidney. Renal ANG I was significantly suppressed in TGR control and untreated HF rats compared to HanSD rats. Both ACEi and ARB treatment significantly raised renal ANG I contents (Fig. 6A). Control TGR rats had renal ANG II levels significantly higher than control HanSD rats. In both rat strains, HF induction caused a significant rise in renal ANG II levels compared to control animals. Rats treated with an ACE inhibitor or AT_1_ blocker had significantly suppressed ANG II concentrations in kidneys (Fig. 6B). Rats treated with an ACE inhibitor had a significantly higher renal ANG I/ANG II ratio than untreated HF rats (Fig. 6C). However, as evident from Figs. 5C and 6C, the extent to which ACEi affected the ANG I/ANG II ratio was remarkably lower in the kidney (approx. 3 × higher in ACEi HanSD and 5 × in ACEi TGR rats compared to controls) than in the plasma (approx. 40 × higher in ACEi HanSD and 50 × in ACEi TGR rats compared to controls). We observed significantly increased renal LDH activity in untreated HF rats compared to control rats. In TGR rats, but not in HanSD rats, there was a slight but significant reduction of elevated LDH activity following ACEi treatment. On the other hand, ARB administration led to a significant reduction in LDH activity in both rat strains (Fig. 6D).

**Figure 7:**
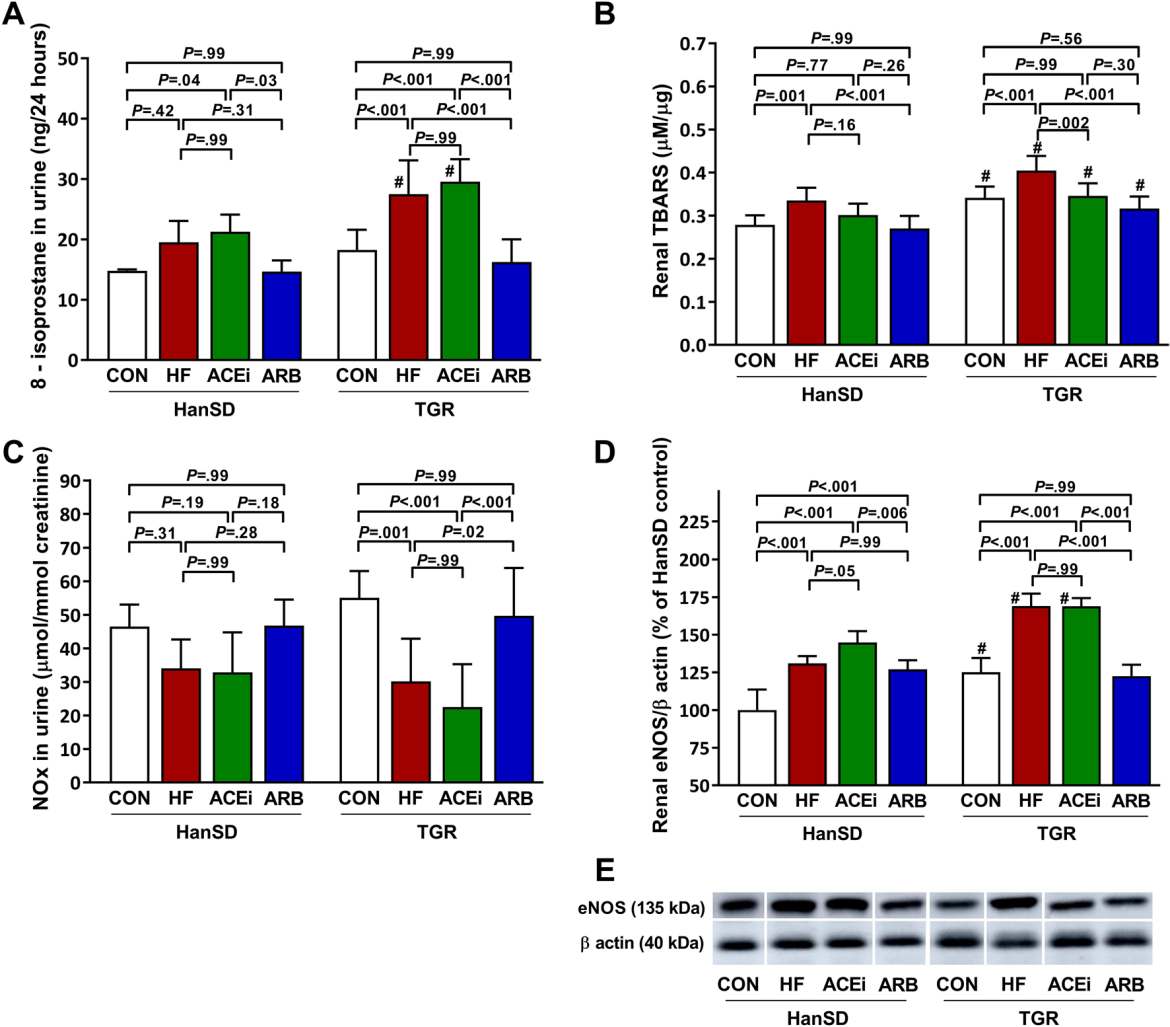
8-isoprostane urine excretion per 24 h (**A**), renal TBARS (**B**), nitrate and nitrite (NOx) urine excretion (**C**) and renal endothelial nitric oxide synthase (eNOS) to β-actin ratios expressed as a percent change from the average of the control HanSD group (**D**) in control (CON), untreated heart failure (HF), ACE inhibitor-treated (ACEi) and AT_1_ receptor blocker-treated (ARB) HanSD and TGR rats 20 weeks after sham or ACF operation (5 weeks in HF TGR rats due to high mortality). Shown in (**E**) are representative western blot images of renal eNOS and β-actin. These images were cropped to improve clarity. All samples were derived from the same experiment and were processed in parallel. Original unprocessed blots can be found in Supplementary Fig. 1. Data presented as mean ± SD (n = 6–9 in each group). ^#^*P* < 0.05 compared to HanSD rats.

Markers of oxidative stress, nitrate and nitrite (NOx) urine excretion, and endothelial nitric oxide synthase (eNOS) expression are presented in Fig. 7. 8-isoprostane in urine and renal thiobarbituric acid reactive substances (TBARS) as markers of oxidative stress are shown in Figs. 7A,B, respectively. We observed a rise in oxidative stress in untreated rats with HF. ACE inhibitor treatment did not significantly influence renal oxidative stress compared to untreated ACF rats of both rat strains, except for ACF TGR rats, in which ACEi significantly reduced renal TBARS. However, HF-induced increase in markers of oxidative stress was ameliorated by blocking the AT_1_ receptor. Urine NOx trended to be lower in untreated HF and ACEi-treated HanSD rats. In TGR rats, urine NOx excretion was significantly reduced in untreated HF and ACEi-treated HanSD rats. In contrast, ARB treatment led to a restoration of NOx excretion (Fig. 7C). Rats with HF showed significantly increased expression of eNOS in the kidney compared to control animals. ACEi adminstration was not associated with significant changes in eNOS expression. In contrast, eNOS expression was significantly suppressed during AT_1_ receptor inhibition in TGR rats (Fig. 7D). Original unprocessed Western blots from Fig. 7E are shown in Supplementary Fig. 1.

## Discussion

Our present study was directly aimed to compare important differences in renal hemodynamics between long-term treatment with an ACE inhibitor or AT_1_ receptor blocker during high-output heart failure. To augment changes seen in normotensive rats, we also performed experiments in rats with ANG II-dependent hypertension. Following ACF induction, a substantial decline in LVEF developed, with mean ejection fraction being 55% in HanSD rats and 54% in TGR rats. Thus, normotensive as well as hypertensive rats with ACF, closely mimic HFpEF. We observed considerably reduced RBF and GFR in rats that underwent the ACF operation. This is in good agreement with previous studies^13,17,18^, and it might explain high mortality noted particularly in TGR rats. Although the administration of an ACE inhibitor for 15 weeks to rats with ACF significantly improved ejection fraction, it did not significantly restore RBF in these rats. Unlike with ACE inhibition, long-term treatment with AT_1_ receptor blocker resulted in significantly improved cardiac functions as well as renal hemodynamics. The involvement of the AT_1_ receptor in RBF regulation in rats with high-output HF was also previously indicated^18,19^. It has been shown that the RAS plays a large role in the RBF decrease in the ACF-based model of HF^13^. However, studies that utilized ACE inhibitors to reduce activated RAS failed to show an improvement in RBF^20,21^. Moreover, our results are in line with that of Duggan and Tabrizchi, who found that in acute settings, only losartan-infused ACF rats responded with an increase in RBF, dissimilar to rats infused with an ACE inhibitor captopril^22^. By contrast, a previous study that utilized the same ACEi treatment as in our study for five weeks in TGR rats with HF improved RBF^14^. Thus, it seems that as HF progresses, the ability of an ACE inhibitor to sufficiently block the RAS in the kidney may decline.

Under physiologic conditions, the kidneys receive approximately 20% of cardiac output. Yet, due to high renal O_2_ demand mainly to drive various transport processes along the nephron, measured O_2_ tensions are surprisingly low, reaching as low as five mmHg in the renal medulla to up to 50 mmHg in the cortex. Therefore, renal parenchyma and especially renal medulla are highly susceptible to hypoxia^23,24^. In our experiment, we showed that untreated and ACEi-treated rats with ACF exhibit significant renal hypoperfusion. That was in untreated rats associated with a GFR reduction, whereas in ACEi-treated rats GFR remained stable. Consequently, hypoxia develops in the kidneys of untreated and ACEi-treated rats with ACF as a result of a mismatch between high oxygen consumption in tubules processing normal quantities of primary urine and reduced O_2_ supply caused by RBF reduction. Indeed, we measured elevated levels of renal LDH activity, as a marker of kidney hypoxia, in untreated and ACEi-treated HF rats (Fig. 6D). Chronic hypoxia promotes tubulointerstitial injury and contributes to a gradual loss of kidney function over time^23–25^. Thus, preventing kidney hypoperfusion and consequently hypoxia by AT_1_ receptor blockade prevents worsening of kidney function. Although not directly measured in our study, it was previously shown that HF induction by ACF placement predisposes to outer medulla injury^26^.

In our experiment HF induction significantly increased renal ANG II content in HanSD rats as well as in transgenic TGR rats with genetically exaggerated RAS activity. AT_1_ receptor blockade significantly reduced renal ANG II levels, suggesting that a major portion of ANG II content in the kidney is of circulatory origin via AT_1_ receptor-mediated uptake. However, an important portion of ANG II is generated in the kidney locally, confirming the importance of intrarenal RAS^27^. Another noteworthy observation is the fact that ACE inhibition did not significantly influence renal ANG II content when compared to AT_1_ receptor blocker-treated rats (Fig. 6B). In certain circumstances, ANG II formation during ACE inhibition is mediated via the activation of specific enzymes such as chymase, cathepsin G and chymostatin-sensitive angiotensin-generating enzyme that can form ANG II independently of ACE. This phenomenon was described previously as “angiotensin II escape”^28^. Inadequate suppression of intrarenal RAS in ACE inhibitor-treated rats with HF might be considered as a plausible explanation for the inability of ACE inhibition to improve RBF significantly. To support this notion, we also examined ANG I/ ANG II ratio as a marker of ACEi efficacy. We demonstrated that in the kidney ACEi affected the ANG I/ANG II ratio remarkably lower than in the plasma (approximately 10x), as evident from Figs. 5C and 6C. Thus, it seems that while plasma ACE is sufficiently inhibited, renal ANG II generation remains at least partly active. Besides intrarenal RAS activation, another important factor contributing to renal hypoperfusion in rats treated with an ACE inhibitor might be the augmented renal vascular responsiveness to ANG II (Fig. 4A,B respectively).

Angiotensin 1–7 is another important peptide of the RAAS cascade involved in the regulation of kidney functions. Angiotensin 1–7 is formed either directly from ANG II in a process involving angiotensin-converting enzyme 2 (ACE2) or indirectly from ANG I with the help of both ACE and ACE2^29^. Angiotensin 1–7 in the kidney mediates its action through the activation of the Mas receptor, which, among other protective actions, leads to vasodilation of renal vasculature (i.e., RBF increase)^30^. In our study, we measured high plasma concentrations of ANG II in rats treated with ARB. It is therefore plausible to assume that in ARB-treated ACF rats ANG II is in greater amounts converted by ACE2 to angiotensin 1–7, which then induces the observed rise in RBF. Increased expression of ACE2 was indeed previously found in kidneys of ACF rats^31^. On the other hand, renal angiotensin 1–7 concentrations in all studied groups of rats in our study were below the limit of detection the ultra-pressure-liquid chromatography-tandem mass spectrometry (LC–MS/MS) method (unpublished results). Therefore, it seems unlikely that the RBF improvement observed in our study in ARB-treated rats is angiotensin 1–7 dependent or angiotensin 1–7 plays just a minor role.

ACF induced heart failure usually leads to a decrease in GFR as observed in our study and previously reported by others^13^. There are several possible mechanisms contributing to GFR reduction. As with RBF decline, the role of excessive RAS activation that occurs during heart failure must be considered. ANG II, the most important peptide of the RAS cascade, constricts both afferent and efferent arteriole via the AT_1_ receptor. However, afferent arteriole is under normal conditions protected from ANG II-induced constriction by counterregulatory systems including the NO system. The inability of the NO system to compensate for vasoconstriction induced by RAS activation in rats with ACF was previously described^18,32^. Thus, in the ACF model of HF, it is plausible to assume that ANG II induces vasoconstriction on both the afferent and efferent arteriole, which leads to a substantial decrease in RBF, associated rise in RVR, and GFR reduction. Nonetheless, the importance of the NO system dysfunction in rats with ACF has been recently questioned^17^. The decrease in GFR can also be attributable to the rise in renal venous pressure. High pressure in renal veins in animals with ACF was previously measured in both acute and chronic settings^33,34^. Fiksen-Olsen and colleagues observed GFR deterioration in response to venous pressure elevation despite normal RBF^35^. The authors argued that once renal venous pressure rises, the subsequent elevation of renal interstitial pressure compresses renal tubules thereby increasing renal tubular pressure that leads to GFR deterioration. Furthermore, the elevation of renal venous pressure causes an increase in renin secretion rate in the kidney and thus intrarenal RAS activation^36^. We suggest that a combination of these mechanisms is responsible for the GFR decrease, as ACF rats without any treatment had high renal venous pressure and renal ANG II content and reduced GFR. After inhibiting the RAS with either ACE inhibitor or AT_1_ blocker, we observed a substantial decrease in the venous pressure and concomitant GFR improvement.

Previous studies suggested that reduced RBF in ACF rats is caused by markedly decreased renal NO bioavailability despite increased renal eNOS levels^18,32^. And that this process is mediated by ANG II through AT_1_ receptor since AT_1_ receptor blockade improved RBF. In our study, we confirmed that renal eNOS expression was indeed increased in rats with ACF, especially in a greater extent in untreated rats and rats treated with ACE inhibitor (Fig. 7D). Lower NO bioavailability was also confirmed by NOx urine excretion in untreated and ACEi-treated ACF rats. These changes were ameliorated when the AT_1_ receptor was blocked. This clearly indicates the involvement of NO availability in renal hemodynamic regulation. The inability to generate NO as a result of HF induction leads to compensatory eNOS overexpression. In addition, acetylcholine, an endothelium-dependent vasodilator, induced smaller RBF increases in untreated rats with ACF compared to control animals. In contrast, when ACE inhibition was present, responses to acetylcholine were substantially augmented (Fig. 4 C,D respectively). This suggests that endothelium is fully capable of vasodilation during ACE inhibition in rats with HF. Yet, RBF does not improve in ACF rats treated with an ACE inhibitor. A variety of causes is associated with decreased NO production including ANG II and ROS generation^37^. Both of these mechanisms might alter NO generation in this HF model. As noted earlier, kidney ANG II levels are elevated in untreated ACF rats and ACEi treatment does not sufficiently prevent ANG II formation and thus action in the kidney. In addition, we observed increased renal levels of oxidative stress products in untreated and ACE inhibitor-treated rats with HF (Fig. 7A,B respectively). Increased oxidative stress in the kidney could also be associated with renal vasoconstriction, enhanced responsiveness to ANG II, bioinactivation of NO and increased renal SNS activity^38,39^.

The SNS is another important system involved in regulation of renal hemodynamics. Increased renal SNS activity enhances RAS activation by renin release and causes renal arteriolar vasoconstriction^40^. We measured increased plasma levels of noradrenaline in rats with ACF, which is in good agreement with previous studies^13,17^. In addition, our data show considerably augmented vascular responses to norepinephrine in ACF rats treated with an ACE inhibitor. In comparison, AT_1_ blocker-treated rats show a decrease in SNS activity. This crosstalk between SNS and RAS might also be responsible for renal vasodilation during high-output heart failure. This assumption is further supported by the fact that inhibition of SNS activity by renal denervation has indeed many protective effects in heart failure^41^.

In our present study, we propose that the improvement of the renal hemodynamics in the ACF model is mediated by these important factors or interactions between them. First, elevated ANG II levels in untreated ACF rats cause vasoconstriction, excessive ROS production and reduced NO production. Similarly, incompletely suppressed ANG II generation by ACEi combined with augmented renal responsiveness to ANG II in ACEi-treated rats did not protect renal hemodynamics. And it seems that only blocking the AT_1_ receptor provides adequate suppression of intrarenal RAS and mitigation of these detrimental effects of ANG II. Second, renal hypoxia further stimulates ROS production^42^, leading to NO bioinactivation and additional vasoconstriction. And third, increased renal SNS activity, especially in ACEi-treated rats, enhances RAS activation and contributes to renal vasoconstriction. We, therefore, suggest that all these factors in kidneys of rats with HF promote renal hypoperfusion. Taken all together, blockade of AT_1_ receptor protected the kidney more efficiently than ACE inhibitor in this model, particularly under the condition of exaggerated RAS activity.

On the other hand, our data on heart weights and dimensions in rats with HF suggest that AT_1_ receptor blockers do not prevent eccentric cardiac hypertrophy as effectively as ACE inhibitors. This is in contrast with the protective effects of AT_1_ blockers against renal hypoperfusion during heart failure. Clinical studies utilizing patients with heart failure showed no statistical difference between ACE inhibitors and AT_1_ blockers on overall mortality. However, in some studies, ACE inhibitor treatment was more effective with regard to the heart function, whereas in others, AT_1_ blockers reduced number of hospitalizations for heart failure^43–45^. Our assumption based on the presented results is that AT_1_ blockers are effective in preventing renal dysfunction in high-output heart failure. On the other hand, ACE inhibitors are more potent in preventing cardiac hypertrophy induced by ACF. This observation is likely caused by the different protective mechanisms that may need further examination in the future. These mechanisms may explain the similar observation in mortality level of ACF rats treated with ACE inhibitor and AT_1_ blocker. Our results may also reopen the discussion on the potential of combined treatment of AT_1_ blockers and ACE inhibitors. However, a large clinical trial that evaluated combined ARB-ACEi treatment in patients at high risk for cardiovascular events reported a significantly higher incidence of adverse effects including hypotension, syncope, kidney dysfunction and hyperkalemia compared to monotherapy^46^. Similarly, a meta-analysis of four studies in patients with left ventricular dysfunction found marked increases in adverse effects in subjects on combination ARB plus ACEi therapy^47^. Although some earlier studies showed mortality and morbidity benefit in patients on dual RAS blockade^48^, in current clinical practice, combination ACE inhibitor-ARB therapy should be avoided^49^. Nonetheless, given the heterogeneity of HFpEF patients, we believe that in a certain subgroup of subjects that suffer from volume overload (e.g., patients with valvular disease, arteriovenous fistulas, severe anemia) combined RAS blockade may be further beneficial. Also, the novel approach that consists of the combined AT_1_ receptor blocker/neprilysin inhibitor treatment needs to be tested to further define the organoprotection of these drugs.

Bearing the murine Ren-2 gene, TGR rats represent a well-established ANG II-dependent model of hypertension^50^. After ACF induction, we observed that time to HF decompensation and eventually death is strikingly shorter as compared to HanSD rats. This was also associated with enhancement of changes seen in HanSD ACF rats (e.g., increase in ROS generation and renal LDH activity, decrease in NO production). Since most of the changes were aggravated in TGR rats, it supports the view that these changes in the ACF model of HF are at least partly ANG II-dependent^18^. On the other hand, spontaneously-hypertensive rats, an ANG II-independent model of hypertension, develop overt hemodynamic signs of high-output HF two weeks after ACF placement, whereas most of these findings were milder or absent in control Wistar rats^51^. Therefore, high blood pressure itself, irrespective of cause, might aggravate the HF development. Of interest are also markedly decreased renal levels of ANG I in control and untreated ACF TGR rats compared to HanSD rats. Together with elevated renal ANG II levels in these rats, it might indicate intrarenal RAS suppression via a negative feedback loop from augmented circulating RAS. Alternatively, intrarenal RAS and especially renal ACE is stimulated by circulating RAS components as suggested in other disease states^27^. Further studies are needed to address this question.

There are several limitations to the study that should be acknowledged. First, we are aware that anesthesia could influence the hemodynamic parameters. This limitation of the present study could be addressed in future studies, which would utilize conscious rats and long-term monitoring of their blood pressure and renal hemodynamics via radiotelemetry device. Also, we did not assess right ventricle parameters. Deleterious effects of right ventricle failure (RVF) on kidney functions were previously proposed^52^. Therefore, future studies should further define the role of RVF in the development of renal dysfunction in this model of HF.

In conclusion, dissimilar to an ACE inhibitor administration, an AT_1_ receptor blocker treatment was shown to prevent renal hypoperfusion and hypoxia in rats with high-output heart failure. Simultaneously, ARB-treated rats exhibited lower ROS generation, improved NO bioavailability, and normal renal SNS activity. The inability of ACE inhibition to improve renal hypoperfusion in ACF rats may result from incomplete intrarenal RAS suppression together with enhanced SNS activity in the face of depleted compensatory mechanisms.

## Materials and methods

As a model of hypertension, male heterozygous rats of the hypertensive Ren-2 transgenic rat (mREN2)27 strain were used^50^. Male HanSD rats served as normotensive counterparts. All animals were bred and housed at the Center of Experimental Medicine of Institute of Clinical and Experimental Medicine, Prague, which is accredited by the Czech Association for Accreditation of Laboratory Animal Care. Rats were housed under standard conditions (12:12 light:dark cycle) and had free access to standard rat chow and water. All animal experiments were approved by the Animal Care and Use Committee of the Institute for Clinical and Experimental Medicine, Prague, in accordance with guidelines and practices established by the Directive 2010/63/EU of the European Parliament on the Protection of Animals Used for Scientific Purposes. A total number of 202 rats (88 HanSD rats and 114 TGR rats) were included in the study, 144 of which were alive at the end of the study protocol and were further analyzed. No power calculation was used to determine the sample size. The sample size was estimated based on the number of successfully analyzed animals in previous experiments in our laboratory. Animals were excluded from the study if successful ACF induction could not be confirmed by inspecting pulsatile flow in the vena cava, death occurred before complete analysis of a given animal, or if a technical error occurred during surgical preparation. The study was carried out in compliance with the ARRIVE guidelines, if not explicitly stated otherwise.

### Heart failure model

HanSD and TGR rats were at the age of eight weeks anesthetized with an intraperitoneal injection of ketamine/midazolam mixture (Calypsol, Gedeon Richter, Hungary, 160 mg/kg and Dormicum, Roche, France, 160 mg/kg). Volume overload-induced heart failure was then induced by creating a shunt between the abdominal aorta and the inferior vena cava using a needle technique. Details of the method were reported by us previously^53^. Briefly, after exposing the abdominal aorta and the inferior vena cava distal of renal arteries a shunt is created between vessels using an 18-gauge needle (1.2 mm in diameter). The puncture wound in the aorta that was created at the time of fistula generation is sealed with cyanoacrylate glue (Histoacryl, B.Braun AG, Germany). Control HanSD and TGR rats underwent a sham operation.

### Experimental protocol and groups

Rats with ACF were left untouched for five weeks for heart failure to fully develop. HanSD rats with ACF were then either left untreated for 15 weeks or received an AT_1_ receptor blocker losartan (Lozap, Zentiva, Prague, CZ) 200 mg/l dissolved in drinking water or an ACE inhibitor trandolapril (Gopten, Mylan, Canonsburg, Pennsylvania, USA) 6 mg/l dissolved in drinking water. Animals were randomized for treatment using a computer-based random group generator. Persons involved in assessing physiological data and biochemical parameters were blinded to treatment allocation. Due to high mortality in TGR rats around the fifth week after ACF operation, these untreated rats were studied at five weeks after surgery. Analogously as HanSD rats, treated TGR rats with ACF also received losartan or trandolapril for 15 weeks after five weeks post ACF placement. Supplementary Fig. 2 summarizes the experimental protocol. There were eight experimental groups in the study, as follows:

- 1. Control HanSD rats studied 20 weeks after sham operation
- 2. Untreated HanSD rats studied 20 weeks after ACF operation
- 3. HanSD rats studied 20 weeks after ACF operation treated with ACE inhibitor
- 4. HanSD rats studied 20 weeks after ACF operation treated with AT_1_ receptor blocker
- 5. Control TGR rats studied 20 weeks after sham operation
- 6. Untreated TGR rats studied five weeks after ACF operation
- 7. TGR rats studied 20 weeks after ACF operation treated with ACE inhibitor
- 8. TGR rats studied 20 weeks after ACF operation treated with AT_1_ receptor blocker

### Echocardiography measurements

Non-invasive assessment of cardiac functions was performed by echocardiography using GE Medical Vivid 7 Dimension System (GE Medical Systems CZ Ltd., Prague, CZ). Rats were prepared according to the experimental protocol (n = 10 in each group) and anesthetized with 2% isoflurane (Abbott Lab, Germany) concentration in inspired air. Standard echocardiograms were recorded by a person blinded to the experiment and analyzed with Echo PAC software (GE Medical Systems CZ Ltd., Prague, CZ). Directly measured parameters from M-mode included left ventricle end-diastolic anterior and posterior wall thickness and left ventricle chamber end-diastolic and end-systolic diameters. Chamber end-systolic volumes (ESV) and end-diastolic volumes (EDV) were derived from the measurements of chamber dimensions by the Teichholz formula as described previously^54^. Ejection fraction (EF) was calculated using formula: EF = (EDV-ESV)/EDV * 100. Heart rate (HR) values were averaged from all evaluated loops.

### Renal hemodynamic studies

Rats from all experimental groups (n = 10 in each group) were prepared according to the protocol described previously for renal hemodynamic studies^14^. Briefly, rats were anesthetized with intraperitoneal thiopental sodium (VUAB Pharma a.s., Prague, CZ) injection at a dose of 80 mg/kg and placed on heated surgical table. Tracheostomy to allow for free breathing was performed and right jugular vein was cannulated. To measure MAP left femoral artery was surgically exposed and cannulated. After opening the abdominal cavity left kidney was surgically freed from the surrounding tissue and placed in a lucite cup. The left ureter was cannulated for urine collection and an ultrasonic transient-time flow probe (1RB, Transonic Systems, Altron Medical Electronic GmbH, Germany) was placed on the left renal artery and RBF was continuously measured. After completion of the surgery a 0.5 ml bolus of an isotonic solution containing 5% sinistrin (Inutest, Fresenius Kabi Austria GmbH, Austria) for GFR measurement and 2% bovine serum albumin (Sigma Chemical Co., Prague, Czech Republic) was given and followed with a continuous infusion of the solution at a rate of 20 µl/min. Rats were allowed to recover from the surgery for 45 min and then urine was collected in three 30 min long periods. A blood sample was obtained from the animals after each collection. Changes in RBF following boluses of vasoactive agents were studies after urine collection was finished. ANG II at a dose of 20 ng and 40 ng, norepinephrine at a dose of 100 ng and 200 ng, and acetylcholine at a dose of 50 ng and 200 ng were given intravenously in a random order. Changes in RBF after the boluses were expressed as a percent change from baseline RBF value to minimum/maximum RBF value after given bolus. Details of this method was recently described^17^. After recovery from the last bolus, mean pressure in the left kidney vein was measured utilizing a technique described by Dong *et al*^55^ using a 2F high-fidelity pressure catheter (Millar Instruments, Houston, Texas, USA). Subsequently, rats were overdosed with a bolus of thiopental given intravenously and organs were weighted.

### Measurements of plasma and renal ANG I, plasma and renal ANG II, markers of oxidative stress, renal LDH activity and eNOS expression

For measurements of plasma and renal ANG I, plasma and renal ANG II, markers of oxidative stress, renal LDH activity and eNOS expression another set of rats from all experimental groups were prepared according to the protocol described previously. At the end of the protocol were rats placed into metabolic cages for 24 h and their urine was collected for further measurements. After a recovery period rats were decapitated, and their plasma and kidneys were rapidly removed and frozen in liquid nitrogen and stored for further analysis.

#### Plasma ANG I and ANG II levels, renal ANG I and ANG II content

Plasma and kidney samples were analyzed by Attoquant Diagnostics GmbH (Vienna, Austria). Plasma ANG I and ANG II and renal ANG I, ANG II, and angiotensin 1–7 levels were measured by LC–MS/MS that provides superior specificity and sensitivity compared to the ELISA method.

#### Plasma noradrenaline

Plasma noradrenaline levels were measured by a solid phase enzyme-linked immunosorbent assay (ELISA) using the commercially available ELISA kit (E4360-100, BioVision, CA, USA).

#### Renal LDH activity

For measurements of renal LDH activity, the commercially available colorimetric assay was utilized (#MAK066, Sigma-Aldrich, St. Louis, MO, USA).

#### 8-isoprostane in urine

8-isoprostane urine concentrations were measured using the commercially available ELISA kit (516,361, Cayman chemical, MI, USA). 8-isoprostane urine concentrations were then multiplied by 24-h urine outputs of rats.

#### Urine nitrate/nitrite

Urine Nitrate/Nitrite levels were measured by a colorimetric assay (780,001, Cayman Chemical, Ann Arbor, MI, USA). Urine creatinine was measured utilizing Liquick Cor-CREATININE kit that is based on modificated Jaffe´s method, without deproteinization (PZ Cormay S.A., Poland). Urine NOx/creatinine ratio was then determined.

#### Renal lipid peroxidation by TBARS assay

TBARS assay was used to determine renal oxidative stress. TBARS levels in kidney samples were analyzed according to our modified method by Shlafer and Shepard^56^. Briefly, a 1:1 mixture of tissue homogenates and 20% (w/v) trichloroacetic acid solution was mixed with a fourfold excess of TBARS reagent (37 mmol/L thiobarbituric acid, 500 mmol/L NaOH, 15% v/v acetic acid) and heated to 100 °C for 70 min. Afterwards, the mixture was left to cool and the pink product was extracted into a 14:1 mixture of 1-butanol/pyridine 535 nm absorbance of which was read by a spectrophotometer (Synergy H1, BioTek, USA). All analyses were performed in duplicate, and their averages were used to calculate the concentration of malondialdehyde (MDA) equivalents in samples according to a calibration curve made from tetrabutylammonium malondialdehyde salt (#63287, Sigma-Aldrich, St. Louis, MO, USA). The final concentration of MDA equivalents in samples was normalized to protein concentration as determined by Bradford assay^57^.

#### Kidney eNOS expression

To determine kidney eNOS expression a ratio between eNOS and β-Actin was determined utilizing SDS-PAGE and western blot analysis. In detail, frozen kidney samples were powdered in liquid nitrogen and subsequently homogenized in SB20 lysis buffer (20% SDS, 10 mmol/L EDTA, 100 mmol/L Tris, pH 6.8). The tissue lysate was diluted in Laemmli sample buffer and boiled for five minutes. An equal amount of protein was loaded in each well and separated by SDS-PAGE on 10% bis-acrylamide gels at a constant voltage of 120 V (Mini-Protean TetraCell, Bio-Rad). After transferring the proteins to a nitrocellulose membrane (0.2 µm pore size, Advantec, Tokyo, Japan) and blocking for four hours with 5% fat-free milk in Tris-buffered saline containing 0.1% Tween 20 (TBST), the membrane was incubated overnight with primary antibodies anti-NOS3 (diluted 1:500, sc-653, Santa Cruz Biotechnology Inc, TX, USA) and anti-β-Actin (diluted 1:5000, A5441, Sigma-Aldrich, St. Louis, MO, USA). The membrane was subsequently washed in TBST and incubated for 1 h with a horseradish peroxidase-linked secondary antibody (diluted 1:2000, #7074/#7076, Cell Signaling Technology, Danvers, MA, USA). The enhanced chemiluminescence method was used for visualization of proteins and the relevant bands were analyzed densitometrically using Carestream Molecular Imaging Software (version 5.0, Carestream Health, New Haven, CT, USA). These techniques are routinely employed at our laboratory^58^. The eNOS expression in the kidney is expressed as a percent change from the average of the control HanSD group.

### Statistical analysis

All values are expressed as means ± standard deviations. GraphPad Prism 8 software (GraphPad software, San Diego, California, USA) was used to perform statistical analysis of the data. Statistical tests included one-way analysis of variance (ANOVA) or two-way ANOVA followed by Bonferroni's multiple comparisons test where appropriate. All results that exceeded 95% probability limits (a p-value lower than 0.05) were reported as statistically significant. Blinding during the data analysis was not considered.

## Data availability

The datasets generated during the current study are publicly available in the figshare repository -https://doi.org/10.6084/m9.figshare.13048598.v1.

## Supplementary Information

Supplementary Information.
