# Supplementary material for: AT1 receptor blocker, but not an ACE inhibitor, prevents kidneys from hypoperfusion during congestive heart failure in normotensive and hypertensive rats
Source: Sci Rep. 2021 Feb 19;11:4271. doi: 10.1038/s41598-021-83906-6 (PMC7896062; doi:10.1038/s41598-021-83906-6)

Supplementary materials for:

AT_1 receptor blocker, but not an ACE inhibitor, prevents kidneys from hypoperfusion during congestive heart failure in normotensive and hypertensive rats

Vojtech Kratky [1,2,3], Zdenka Vanourkova [1], Matus Sykora [4], Barbara Szeiffova Bacova[4], Zdenka Hruskova [3], Sona Kikerlova [1], Zuzana Huskova [1], Libor Kopkan [1]

[1] Center for Experimental Medicine, Institute for Clinical and Experimental Medicine, Prague, Czech Republic.

[2] Department of Pathophysiology, 2nd Faculty of Medicine, Charles University, Prague, Czech Republic.

[3] Department of Nephrology, First Faculty of Medicine, Charles University and General University Hospital in Prague, Prague, Czech Republic

[4] Institute for Heart Research, Centre of Experimental Medicine, Slovak Academy of Sciences, Bratislava, Slovak republic

Author for correspondence:

Vojtech Kratky, M.D.

Center for Experimental Medicine, Institute for Clinical and Experimental Medicine, 1958/9 Videnska, CZ-140 00 Prague 4, Czech Republic.

Email: Vojtech.Kratky@vfn.cz

Supplementary Table 1 Organ weights of control, untreated heart failure (HF), ACE inhibitor-treated (HF + ACEi) and AT_1 receptor blocker-treated (HF + ARB) HanSD and TGR rats. Please note that data from HF TGR rats were obtain in 5 weeks after ACF induction due to high mortality.

	**HanSD rats**				**TGR rats**			
	Control	HF	HF + ACEi	HF + ARB	Control	HF	HF + ACEi	HF + ARB
Heart weight (mg)	1543 ± 115	2782 ± 481 *	2306 ± 196 * #	2514 ± 256 *	1950 ± 131 ¥	2468 ± 337 *	2149 ± 257 * #	2202 ± 272 * #
Left ventricle weight (mg)	1108 ± 78	1760 ± 232 *	1527 ± 114 * #	1660 ± 147 *	1482 ± 85 ¥	1687 ± 159 *	1418 ± 157 # ¥	1481 ± 178 # ¥
Right ventricle weight (mg)	309 ± 31	625 ± 126 *	522 ± 54 * #	572 ± 76 *	315 ± 30 *	490 ± 106 *	519 ± 124 *	492 ± 75 *
Lung weight (mg)	1987 ± 126	2578 ± 623 *	2216 ± 216 #	2278 ± 172 #	2057 ± 182	2558 ± 638 *	2333 ± 235 #	2331 ± 171 #
Liver weight (g)	16.8 ± 2.0	18.3 ± 7.2	17.1 ± 2.5	16.8 ± 2.1	20.0 ± 1.3	17.1 ± 3.5	17.8 ± 2.7	19.1 ± 2.3
Kidney weight (mg)	1933 ± 182	1801 ± 229	1918 ± 233	1887 ± 170	2134 ± 76	1740 ± 164	1863 ± 227	1984 ± 153

Values are expressed as mean ± standard deviation and consist of ten observations per group. * $p < 0.05$ versus control of the same rat strain # $p < 0.05$ versus HF of the same rat strain ¥ $p < 0.05$ versus HanSD counterparts.

Supplementary Figure 1. Original unprocessed Western blots from main Figure 7E.

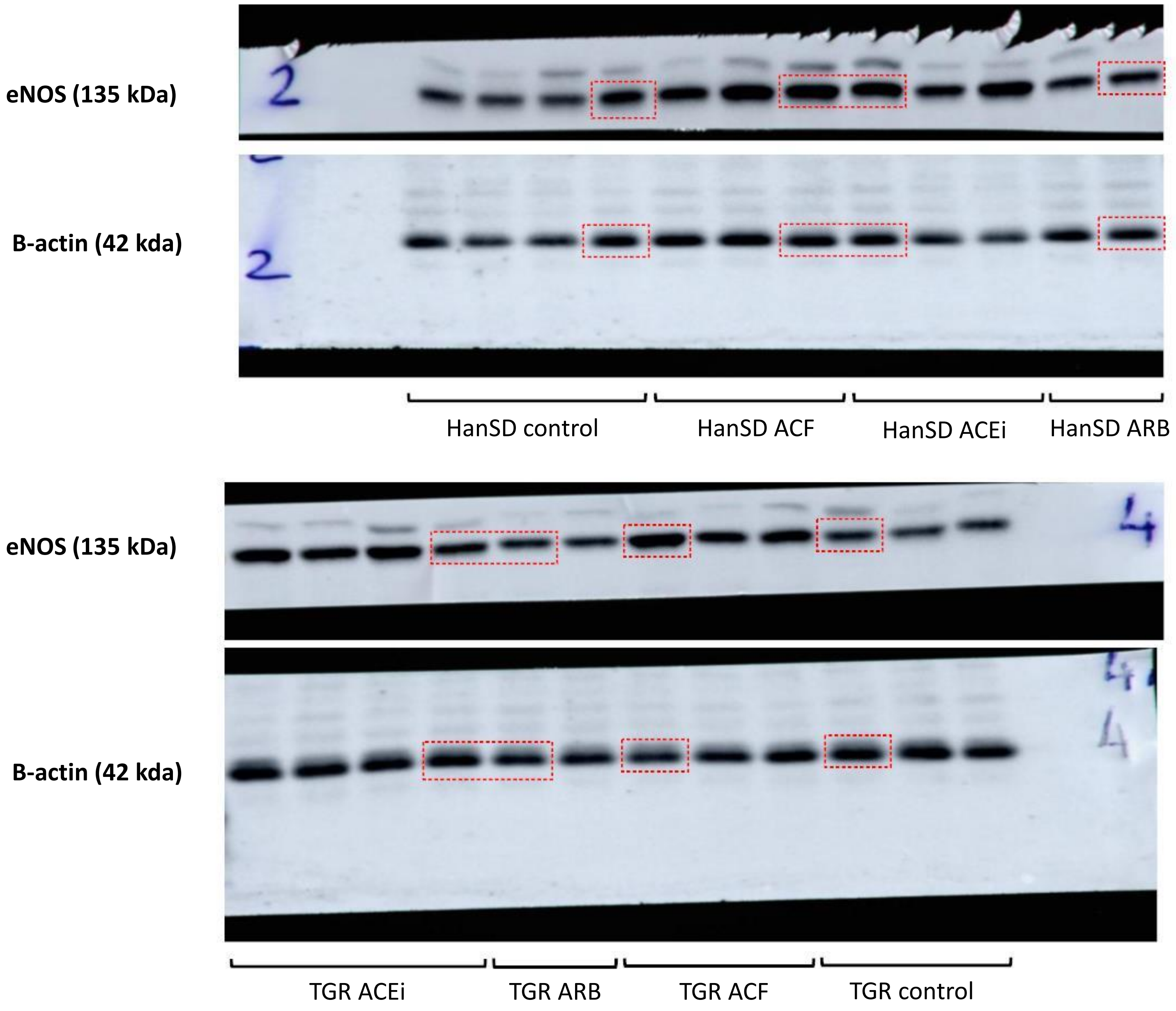

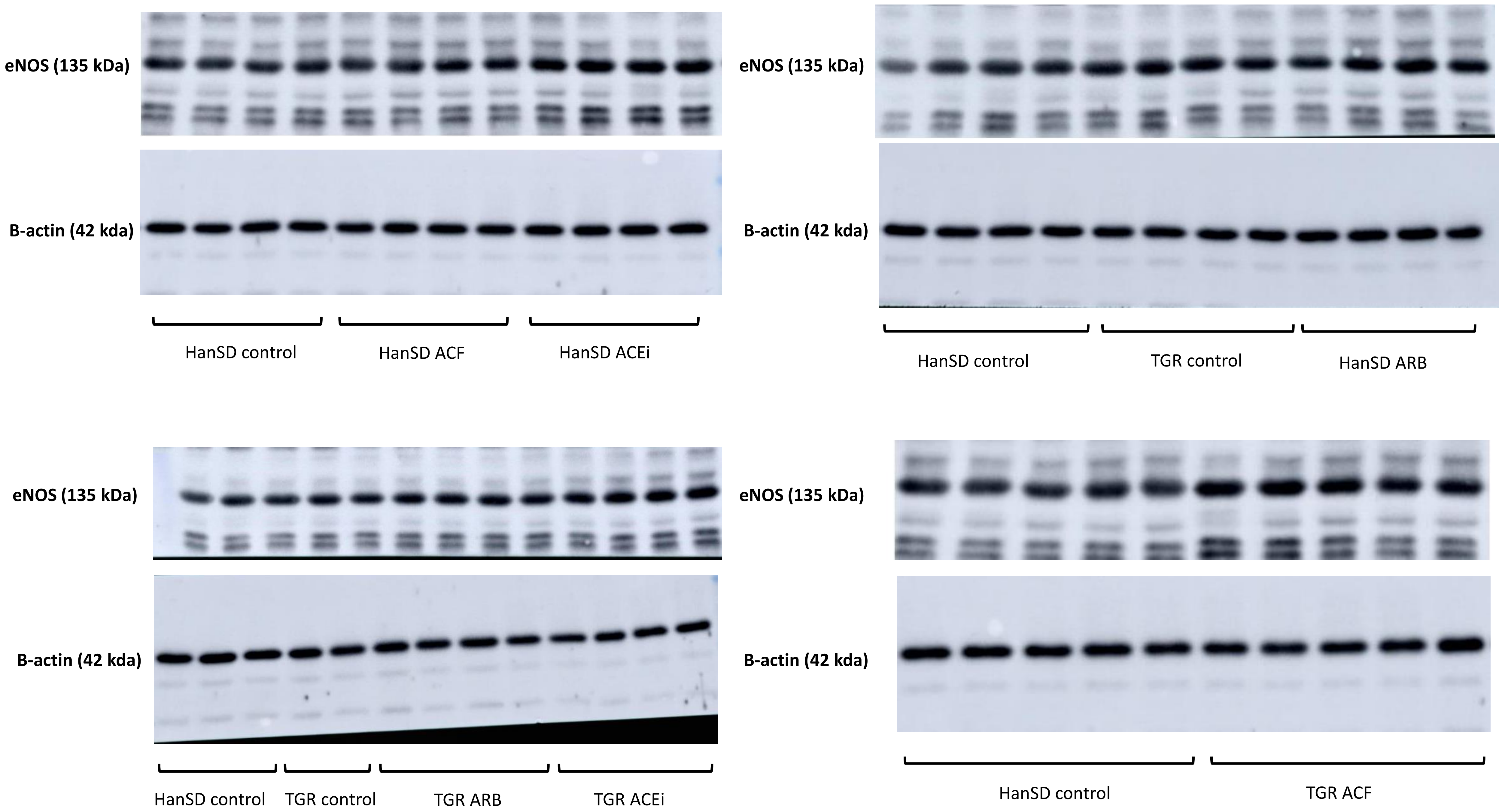
eNOS (135 kDa)
B-actin (42 kda)
HanSD control
HanSD ACF
HanSD ACEi
eNOS (135 kDa)
B-actin (42 kda)
HanSD control
TGR control
HanSD ARB
eNOS (135 kDa)
B-actin (42 kda)
HanSD control
TGR control
TGR ARB
TGR ACEi
eNOS (135 kDa)
B-actin (42 kda)
HanSD control
TGR ACF

Supplementary Figure 2. Experimental protocol

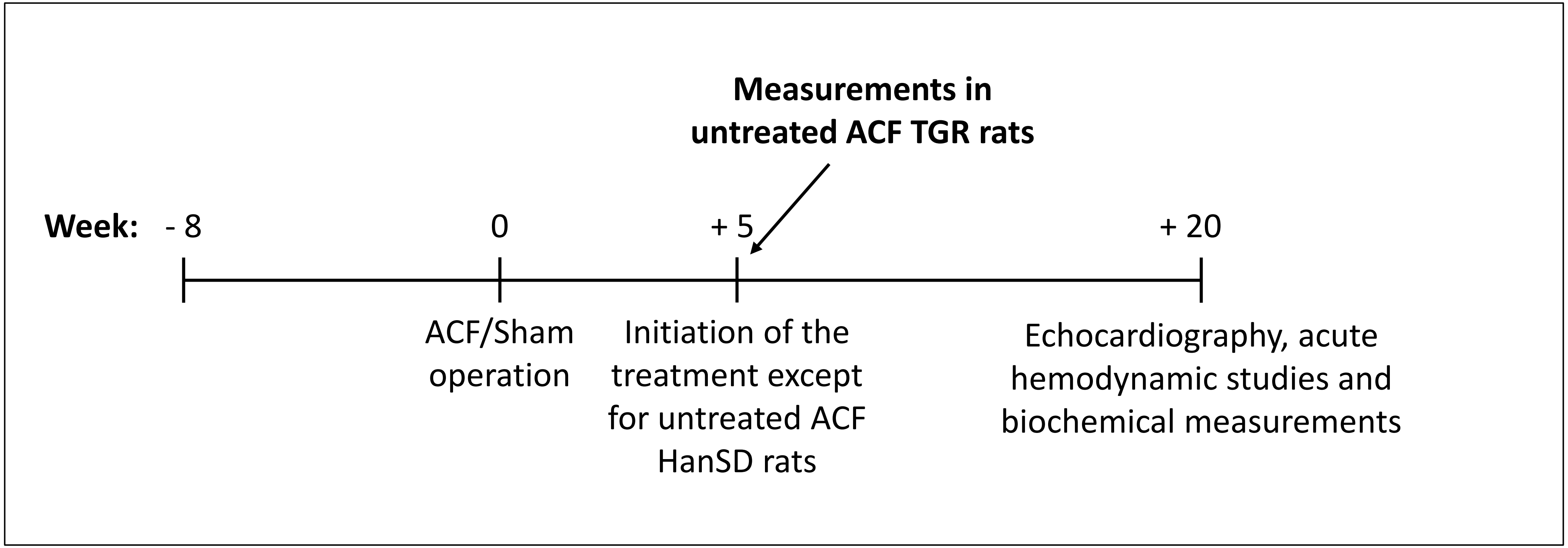

Supplement: Supplementary file 1 — Supplementary Information. [file 41598_2021_83906_MOESM1_ESM.pdf]
